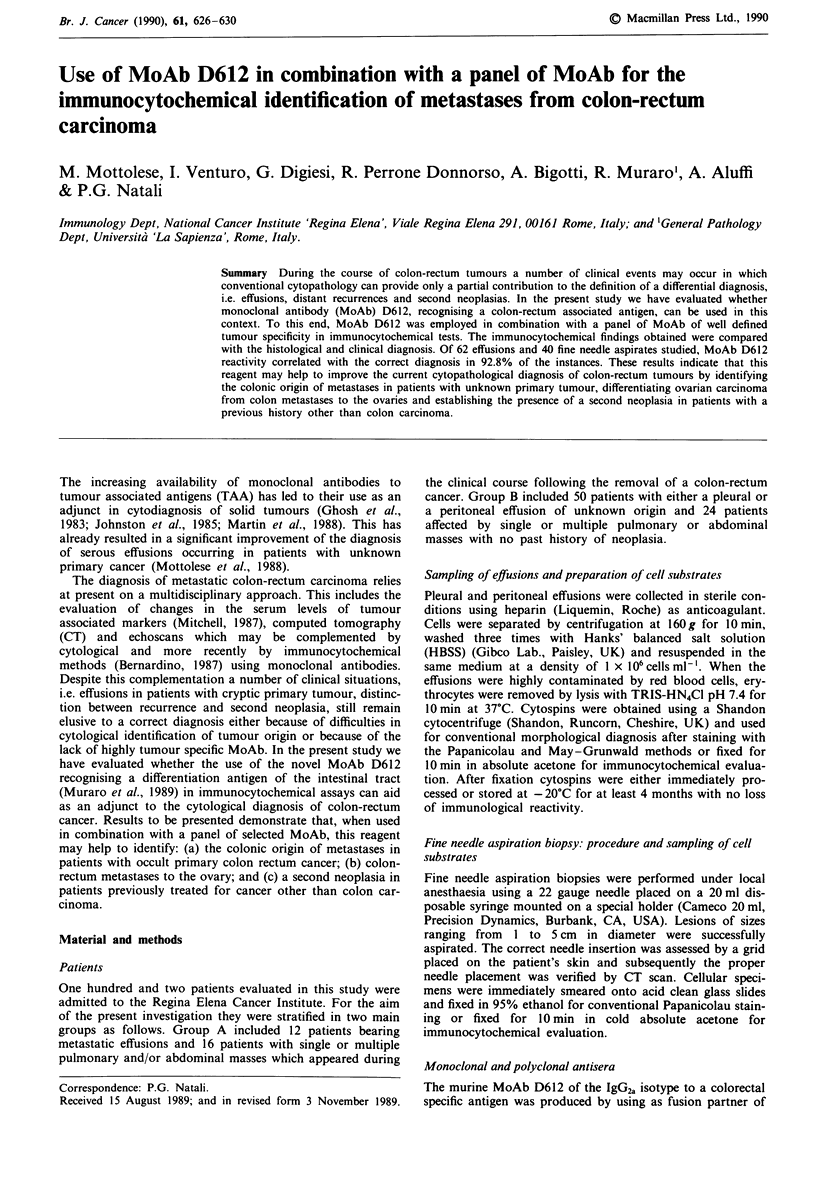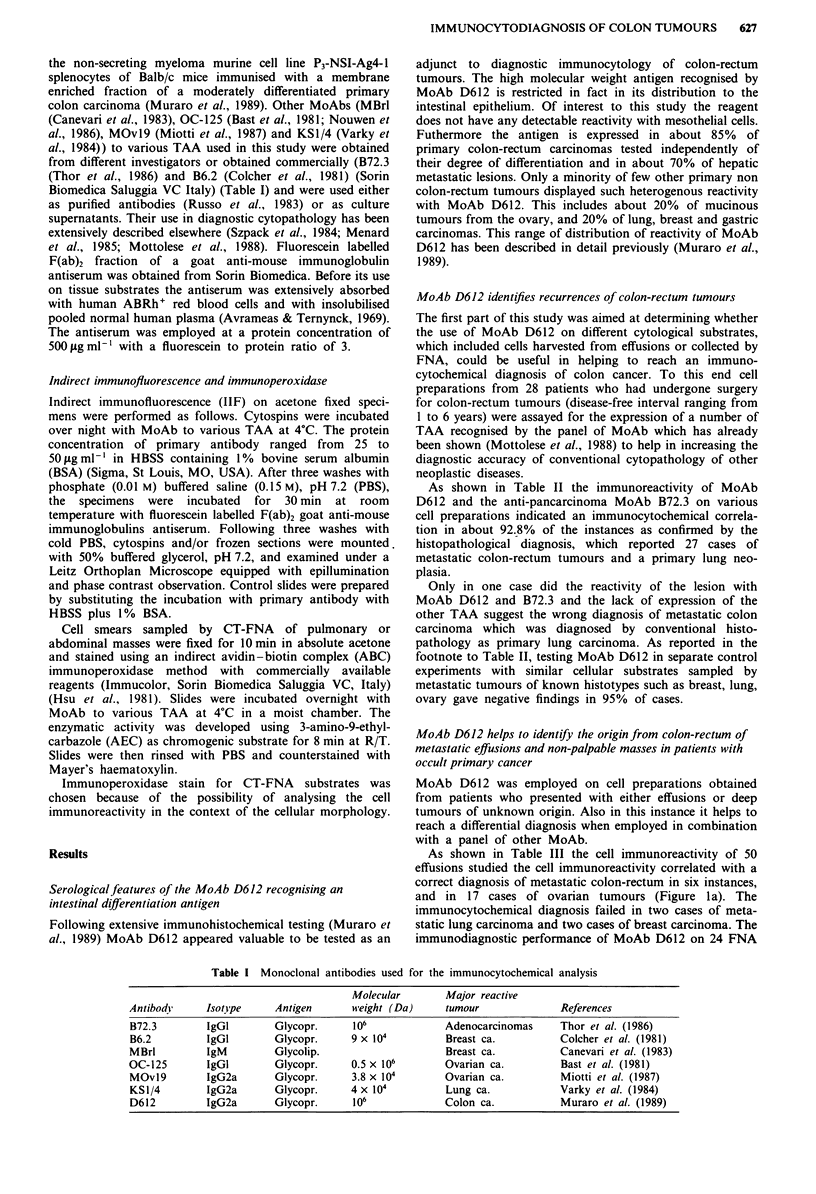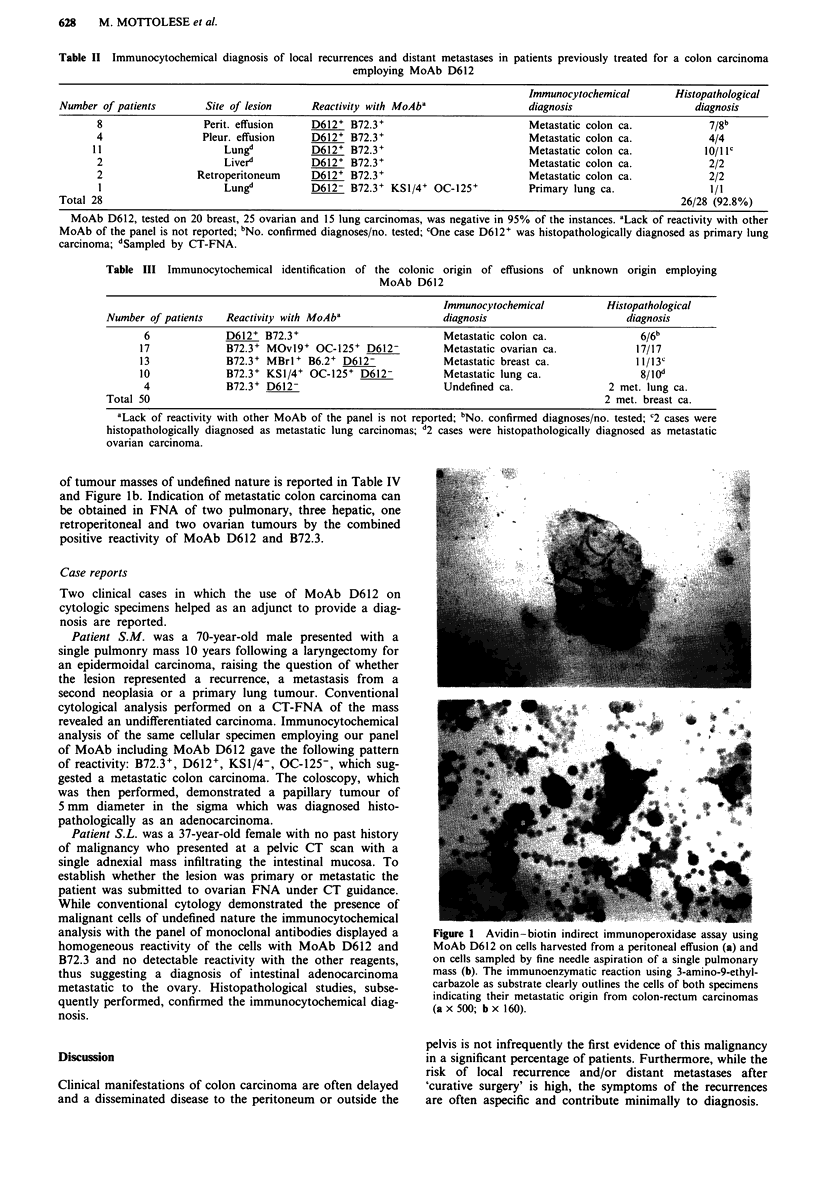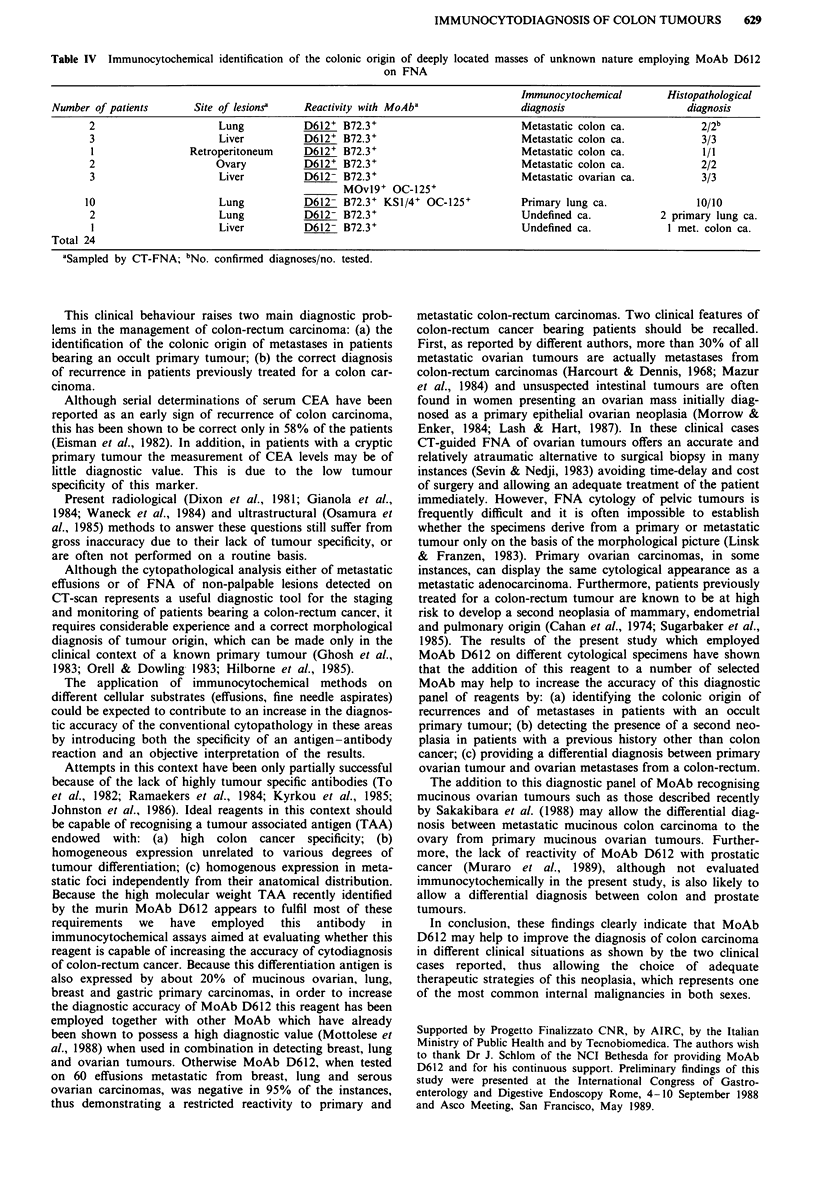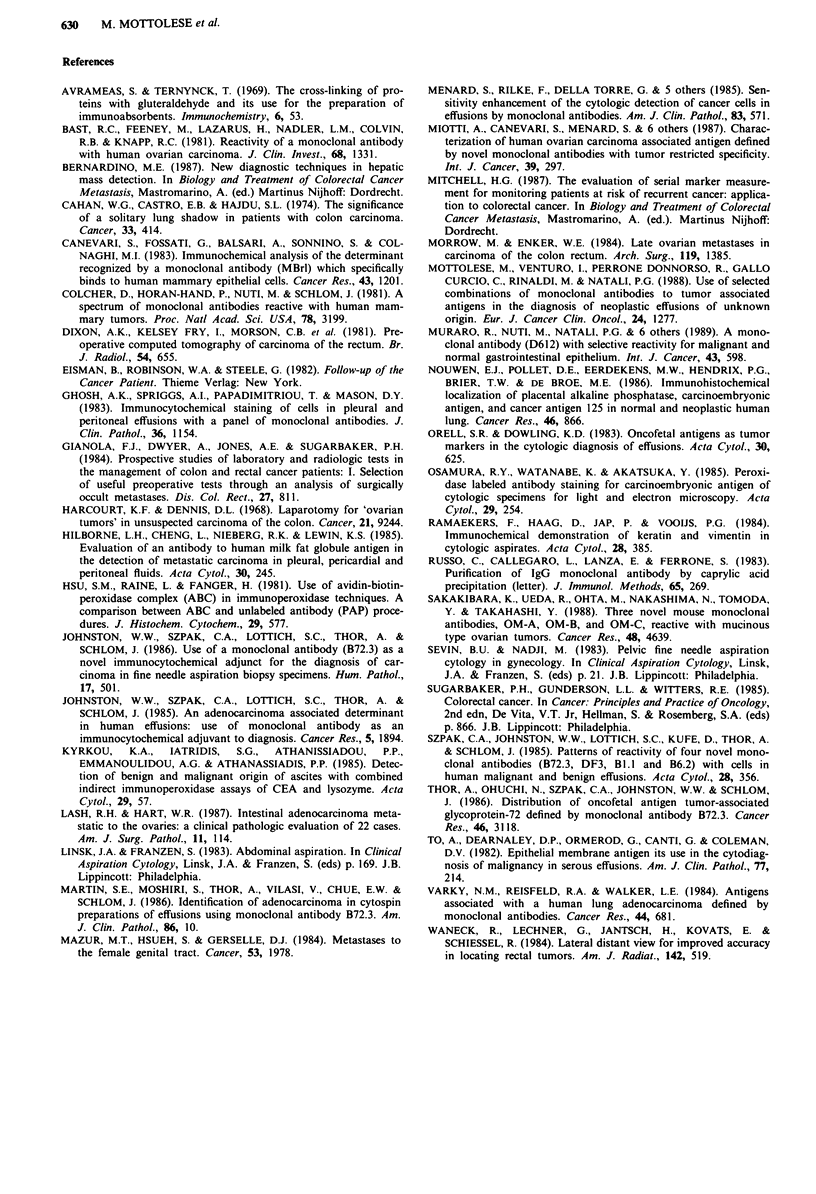# Use of MoAb D612 in combination with a panel of MoAb for the immunocytochemical identification of metastases from colon-rectum carcinoma.

**DOI:** 10.1038/bjc.1990.139

**Published:** 1990-04

**Authors:** M. Mottolese, I. Venturo, G. Digiesi, R. Perrone Donnorso, A. Bigotti, R. Muraro, A. Aluffi, P. G. Natali

**Affiliations:** Immunology Dept, National Cancer Institute Regina Elena, Rome, Italy.

## Abstract

**Images:**


Br. J. Cancer (1990), 61, 626-630 © Macmillan Press Ltd., 1990

### Use of MoAb D612 in combination with a panel of MoAb for the immunocytochemical identification of metastases from colon-rectum carcinoma

M. Mottolese, I. Venturo, G. Digiesi, R. Perrone Donnorso, A. Bigotti, R. Muraro[1], A. Aluffi & P.G. Natali

*Immunology Dept, National Cancer Institute 'Regina Elena', Viale Regina Elena 291, 00161 Rome, Italy; and [1]General Pathology Dept, Università 'La Sapienza', Rome, Italy.*

**Summary** During the course of colon-rectum tumours a number of clinical events may occur in which conventional cytopathology can provide only a partial contribution to the definition of a differential diagnosis, i.e. effusions, distant recurrences and second neoplasias. In the present study we have evaluated whether monoclonal antibody (MoAb) D612, recognising a colon-rectum associated antigen, can be used in this context. To this end, MoAb D612 was employed in combination with a panel of MoAb of well defined tumour specificity in immunocytochemical tests. The immunocytochemical findings obtained were compared with the histological and clinical diagnosis. Of 62 effusions and 40 fine needle aspirates studied, MoAb D612 reactivity correlated with the correct diagnosis in 92.8% of the instances. These results indicate that this reagent may help to improve the current cytopathological diagnosis of colon-rectum tumours by identifying the colonic origin of metastases in patients with unknown primary tumour, differentiating ovarian carcinoma from colon metastases to the ovaries and establishing the presence of a second neoplasia in patients with a previous history other than colon carcinoma.

The increasing availability of monoclonal antibodies to tumour associated antigens (TAA) has led to their use as an adjunct in cytodiagnosis of solid tumours (Ghosh *et al.*, 1983; Johnston *et al.*, 1985; Martin *et al.*, 1988). This has already resulted in a significant improvement of the diagnosis of serous effusions occurring in patients with unknown primary cancer (Mottolese *et al.*, 1988).

The diagnosis of metastatic colon-rectum carcinoma relies at present on a multidisciplinary approach. This includes the evaluation of changes in the serum levels of tumour associated markers (Mitchell, 1987), computed tomography (CT) and echoscans which may be complemented by cytological and more recently by immunocytochemical methods (Bernardino, 1987) using monoclonal antibodies. Despite this complementation a number of clinical situations, i.e. effusions in patients with cryptic primary tumour, distinction between recurrence and second neoplasia, still remain elusive to a correct diagnosis either because of difficulties in cytological identification of tumour origin or because of the lack of highly tumour specific MoAb. In the present study we have evaluated whether the use of the novel MoAb D612 recognising a differentiation antigen of the intestinal tract (Muraro *et al.*, 1989) in immunocytochemical assays can aid as an adjunct to the cytological diagnosis of colon-rectum cancer. Results to be presented demonstrate that, when used in combination with a panel of selected MoAb, this reagent may help to identify: (a) the colonic origin of metastases in patients with occult primary colon rectum cancer; (b) colon-rectum metastases to the ovary; and (c) a second neoplasia in patients previously treated for cancer other than colon carcinoma.

### Material and methods

### *Patients*

One hundred and two patients evaluated in this study were admitted to the Regina Elena Cancer Institute. For the aim of the present investigation they were stratified in two main groups as follows. Group A included 12 patients bearing metastatic effusions and 16 patients with single or multiple pulmonary and/or abdominal masses which appeared during the clinical course following the removal of a colon-rectum cancer. Group B included 50 patients with either a pleural or a peritoneal effusion of unknown origin and 24 patients affected by single or multiple pulmonary or abdominal masses with no past history of neoplasia.

### *Sampling of effusions and preparation of cell substrates*

Pleural and peritoneal effusions were collected in sterile conditions using heparin (Liquemin, Roche) as anticoagulant. Cells were separated by centrifugation at 160 *g* for 10 min, washed three times with Hanks' balanced salt solution (HBSS) (Gibco Lab., Paisley, UK) and resuspended in the same medium at a density of $1 \times 10^6$ cells $ml^{-1}$. When the effusions were highly contaminated by red blood cells, erythrocytes were removed by lysis with TRIS-$HN_4Cl$ pH 7.4 for 10 min at 37°C. Cytospins were obtained using a Shandon cytocentrifuge (Shandon, Runcorn, Cheshire, UK) and used for conventional morphological diagnosis after staining with the Papanicolau and May-Grunwald methods or fixed for 10 min in absolute acetone for immunocytochemical evaluation. After fixation cytospins were either immediately processed or stored at −20°C for at least 4 months with no loss of immunological reactivity.

### *Fine needle aspiration biopsy: procedure and sampling of cell substrates*

Fine needle aspiration biopsies were performed under local anesthaesia using a 22 gauge needle placed on a 20 ml disposable syringe mounted on a special holder (Cameco 20 ml, Precision Dynamics, Burbank, CA, USA). Lesions of sizes ranging from 1 to 5 cm in diameter were successfully aspirated. The correct needle insertion was assessed by a grid placed on the patient's skin and subsequently the proper needle placement was verified by CT scan. Cellular specimens were immediately smeared onto acid clean glass slides and fixed in 95% ethanol for conventional Papanicolau staining or fixed for 10 min in cold absolute acetone for immunocytochemical evaluation.

### *Monoclonal and polyclonal antisera*

The murine MoAb D612 of the $IgG_{2a}$ isotype to a colorectal specific antigen was produced by using as fusion partner of

Correspondence: P.G. Natali.
Received 15 August 1989; and in revised form 3 November 1989.

the non-secreting myeloma murine cell line $P_3$-NSI-Ag4-1 splenocytes of Balb/c mice immunised with a membrane enriched fraction of a moderately differentiated primary colon carcinoma (Muraro *et al.*, 1989). Other MoAbs (MBrl (Canevari *et al.*, 1983), OC-125 (Bast *et al.*, 1981; Nouwen *et al.*, 1986), MOv19 (Miotti *et al.*, 1987) and KS1/4 (Varky *et al.*, 1984)) to various TAA used in this study were obtained from different investigators or obtained commercially (B72.3 (Thor *et al.*, 1986) and B6.2 (Colcher *et al.*, 1981) (Sorin Biomedica Saluggia VC Italy) (Table I) and were used either as purified antibodies (Russo *et al.*, 1983) or as culture supernatants. Their use in diagnostic cytopathology has been extensively described elsewhere (Szpack *et al.*, 1984; Menard *et al.*, 1985; Mottolese *et al.*, 1988). Fluorescein labelled $F(ab)_2$ fraction of a goat anti-mouse immunoglobulin antiserum was obtained from Sorin Biomedica. Before its use on tissue substrates the antiserum was extensively absorbed with human $ABRh^+$ red blood cells and with insolubilised pooled normal human plasma (Avrameas & Ternynck, 1969). The antiserum was employed at a protein concentration of 500 $\mu g\, ml^{-1}$ with a fluorescein to protein ratio of 3.

*Indirect immunofluorescence and immunoperoxidase*

Indirect immunofluorescence (IIF) on acetone fixed specimens were performed as follows. Cytospins were incubated over night with MoAb to various TAA at 4°C. The protein concentration of primary antibody ranged from 25 to 50 $\mu g\, ml^{-1}$ in HBSS containing 1% bovine serum albumin (BSA) (Sigma, St Louis, MO, USA). After three washes with phosphate (0.01 M) buffered saline (0.15 M), pH 7.2 (PBS), the specimens were incubated for 30 min at room temperature with fluorescein labelled $F(ab)_2$ goat anti-mouse immunoglobulins antiserum. Following three washes with cold PBS, cytospins and/or frozen sections were mounted with 50% buffered glycerol, pH 7.2, and examined under a Leitz Orthoplan Microscope equipped with epillumination and phase contrast observation. Control slides were prepared by substituting the incubation with primary antibody with HBSS plus 1% BSA.

Cell smears sampled by CT-FNA of pulmonary or abdominal masses were fixed for 10 min in absolute acetone and stained using an indirect avidin-biotin complex (ABC) immunoperoxidase method with commercially available reagents (Immucolor, Sorin Biomedica Saluggia VC, Italy) (Hsu *et al.*, 1981). Slides were incubated overnight with MoAb to various TAA at 4°C in a moist chamber. The enzymatic activity was developed using 3-amino-9-ethylcarbazole (AEC) as chromogenic substrate for 8 min at R/T. Slides were then rinsed with PBS and counterstained with Mayer's haematoxylin.

Immunoperoxidase stain for CT-FNA substrates was chosen because of the possibility of analysing the cell immunoreactivity in the context of the cellular morphology.

### Results

*Serological features of the MoAb D612 recognising an intestinal differentiation antigen*

Following extensive immunohistochemical testing (Muraro *et al.*, 1989) MoAb D612 appeared valuable to be tested as an adjunct to diagnostic immunocytology of colon-rectum tumours. The high molecular weight antigen recognised by MoAb D612 is restricted in fact in its distribution to the intestinal epithelium. Of interest to this study the reagent does not have any detectable reactivity with mesothelial cells. Futhermore the antigen is expressed in about 85% of primary colon-rectum carcinomas tested independently of their degree of differentiation and in about 70% of hepatic metastatic lesions. Only a minority of few other primary non colon-rectum tumours displayed such heterogenous reactivity with MoAb D612. This includes about 20% of mucinous tumours from the ovary, and 20% of lung, breast and gastric carcinomas. This range of distribution of reactivity of MoAb D612 has been described in detail previously (Muraro *et al.*, 1989).

*MoAb D612 identifies recurrences of colon-rectum tumours*

The first part of this study was aimed at determining whether the use of MoAb D612 on different cytological substrates, which included cells harvested from effusions or collected by FNA, could be useful in helping to reach an immunocytochemical diagnosis of colon cancer. To this end cell preparations from 28 patients who had undergone surgery for colon-rectum tumours (disease-free interval ranging from 1 to 6 years) were assayed for the expression of a number of TAA recognised by the panel of MoAb which has already been shown (Mottolese *et al.*, 1988) to help in increasing the diagnostic accuracy of conventional cytopathology of other neoplastic diseases.

As shown in Table II the immunoreactivity of MoAb D612 and the anti-pancarcinoma MoAb B72.3 on various cell preparations indicated an immunocytochemical correlation in about 92.8% of the instances as confirmed by the histopathological diagnosis, which reported 27 cases of metastatic colon-rectum tumours and a primary lung neoplasia.

Only in one case did the reactivity of the lesion with MoAb D612 and B72.3 and the lack of expression of the other TAA suggest the wrong diagnosis of metastatic colon carcinoma which was diagnosed by conventional histopathology as primary lung carcinoma. As reported in the footnote to Table II, testing MoAb D612 in separate control experiments with similar cellular substrates sampled by metastatic tumours of known histotypes such as breast, lung, ovary gave negative findings in 95% of cases.

*MoAb D612 helps to identify the origin from colon-rectum of metastatic effusions and non-palpable masses in patients with occult primary cancer*

MoAb D612 was employed on cell preparations obtained from patients who presented with either effusions or deep tumours of unknown origin. Also in this instance it helps to reach a differential diagnosis when employed in combination with a panel of other MoAb.

As shown in Table III the cell immunoreactivity of 50 effusions studied the cell immunoreactivity correlated with a correct diagnosis of metastatic colon-rectum in six instances, and in 17 cases of ovarian tumours (Figure 1a). The immunocytochemical diagnosis failed in two cases of metastatic lung carcinoma and two cases of breast carcinoma. The immunodiagnostic performance of MoAb D612 on 24 FNA

**Table I** Monoclonal antibodies used for the immunocytochemical analysis

| *Antibody* | *Isotype* | *Antigen* | *Molecular weight (Da)* | *Major reactive tumour* | *References* |
|---|---|---|---|---|---|
| B72.3 | IgGl | Glycopr. | $10^6$ | Adenocarcinomas | Thor *et al.* (1986) |
| B6.2 | IgGl | Glycopr. | $9 \times 10^4$ | Breast ca. | Colcher *et al.* (1981) |
| MBrl | IgM | Glycolip. | | Breast ca. | Canevari *et al.* (1983) |
| OC-125 | IgGl | Glycopr. | $0.5 \times 10^6$ | Ovarian ca. | Bast *et al.* (1981) |
| MOv19 | IgG2a | Glycopr. | $3.8 \times 10^4$ | Ovarian ca. | Miotti *et al.* (1987) |
| KS1/4 | IgG2a | Glycopr. | $4 \times 10^4$ | Lung ca. | Varky *et al.* (1984) |
| D612 | IgG2a | Glycopr. | $10^6$ | Colon ca. | Muraro *et al.* (1989) |

**Table II** Immunocytochemical diagnosis of local recurrences and distant metastases in patients previously treated for a colon carcinoma employing MoAb D612

| *Number of patients* | *Site of lesion* | *Reactivity with MoAb*[a] | *Immunocytochemical diagnosis* | *Histopathological diagnosis* |
|---|---|---|---|---|
| 8 | Perit. effusion | D612+ B72.3+ | Metastatic colon ca. | 7/8[b] |
| 4 | Pleur. effusion | D612+ B72.3+ | Metastatic colon ca. | 4/4 |
| 11 | Lung[d] | D612+ B72.3+ | Metastatic colon ca. | 10/11[c] |
| 2 | Liver[d] | D612+ B72.3+ | Metastatic colon ca. | 2/2 |
| 2 | Retroperitoneum | D612+ B72.3+ | Metastatic colon ca. | 2/2 |
| 1 | Lung[d] | D612− B72.3+ KS1/4+ OC-125+ | Primary lung ca. | 1/1 |
| Total 28 | | | | 26/28 (92.8%) |

MoAb D612, tested on 20 breast, 25 ovarian and 15 lung carcinomas, was negative in 95% of the instances. [a]Lack of reactivity with other MoAb of the panel is not reported; [b]No. confirmed diagnoses/no. tested; [c]One case D612+ was histopathologically diagnosed as primary lung carcinoma; [d]Sampled by CT-FNA.

**Table III** Immunocytochemical identification of the colonic origin of effusions of unknown origin employing MoAb D612

| *Number of patients* | *Reactivity with MoAb*[a] | *Immunocytochemical diagnosis* | *Histopathological diagnosis* |
|---|---|---|---|
| 6 | D612+ B72.3+ | Metastatic colon ca. | 6/6[b] |
| 17 | B72.3+ MOv19+ OC-125+ D612− | Metastatic ovarian ca. | 17/17 |
| 13 | B72.3+ MBr1+ B6.2+ D612− | Metastatic breast ca. | 11/13[c] |
| 10 | B72.3+ KS1/4+ OC-125+ D612− | Metastatic lung ca. | 8/10[d] |
| 4 | B72.3+ D612− | Undefined ca. | 2 met. lung ca. |
| Total 50 | | | 2 met. breast ca. |

[a]Lack of reactivity with other MoAb of the panel is not reported; [b]No. confirmed diagnoses/no. tested; [c]2 cases were histopathologically diagnosed as metastatic lung carcinomas; [d]2 cases were histopathologically diagnosed as metastatic ovarian carcinoma.

of tumour masses of undefined nature is reported in Table IV and Figure 1b. Indication of metastatic colon carcinoma can be obtained in FNA of two pulmonary, three hepatic, one retroperitoneal and two ovarian tumours by the combined positive reactivity of MoAb D612 and B72.3.

*Case reports*

Two clinical cases in which the use of MoAb D612 on cytologic specimens helped as an adjunct to provide a diagnosis are reported.

*Patient S.M.* was a 70-year-old male presented with a single pulmonry mass 10 years following a laryngectomy for an epidermoidal carcinoma, raising the question of whether the lesion represented a recurrence, a metastasis from a second neoplasia or a primary lung tumour. Conventional cytological analysis performed on a CT-FNA of the mass revealed an undifferentiated carcinoma. Immunocytochemical analysis of the same cellular specimen employing our panel of MoAb including MoAb D612 gave the following pattern of reactivity: B72.3+, D612+, KS1/4−, OC-125−, which suggested a metastatic colon carcinoma. The coloscopy, which was then performed, demonstrated a papillary tumour of 5 mm diameter in the sigma which was diagnosed histopathologically as an adenocarcinoma.

*Patient S.L.* was a 37-year-old female with no past history of malignancy who presented at a pelvic CT scan with a single adnexial mass infiltrating the intestinal mucosa. To establish whether the lesion was primary or metastatic the patient was submitted to ovarian FNA under CT guidance. While conventional cytology demonstrated the presence of malignant cells of undefined nature the immunocytochemical analysis with the panel of monoclonal antibodies displayed a homogeneous reactivity of the cells with MoAb D612 and B72.3 and no detectable reactivity with the other reagents, thus suggesting a diagnosis of intestinal adenocarcinoma metastatic to the ovary. Histopathological studies, subsequently performed, confirmed the immunocytochemical diagnosis.

**Figure 1** Avidin–biotin indirect immunoperoxidase assay using MoAb D612 on cells harvested from a peritoneal effusion (**a**) and on cells sampled by fine needle aspiration of a single pulmonary mass (**b**). The immunoenzymatic reaction using 3-amino-9-ethylcarbazole as substrate clearly outlines the cells of both specimens indicating their metastatic origin from colon-rectum carcinomas (**a** × 500; **b** × 160).

### Discussion

Clinical manifestations of colon carcinoma are often delayed and a disseminated disease to the peritoneum or outside the pelvis is not infrequently the first evidence of this malignancy in a significant percentage of patients. Furthermore, while the risk of local recurrence and/or distant metastases after 'curative surgery' is high, the symptoms of the recurrences are often aspecific and contribute minimally to diagnosis.

**Table IV** Immunocytochemical identification of the colonic origin of deeply located masses of unknown nature employing MoAb D612 on FNA

| *Number of patients* | *Site of lesions*[a] | *Reactivity with MoAb*[a] | *Immunocytochemical diagnosis* | *Histopathological diagnosis* |
|---|---|---|---|---|
| 2 | Lung | D612$^+$ B72.3$^+$ | Metastatic colon ca. | 2/2[b] |
| 3 | Liver | D612$^+$ B72.3$^+$ | Metastatic colon ca. | 3/3 |
| 1 | Retroperitoneum | D612$^+$ B72.3$^+$ | Metastatic colon ca. | 1/1 |
| 2 | Ovary | D612$^+$ B72.3$^+$ | Metastatic colon ca. | 2/2 |
| 3 | Liver | D612$^-$ B72.3$^+$ MOv19$^+$ OC-125$^+$ | Metastatic ovarian ca. | 3/3 |
| 10 | Lung | D612$^-$ B72.3$^+$ KS1/4$^+$ OC-125$^+$ | Primary lung ca. | 10/10 |
| 2 | Lung | D612$^-$ B72.3$^+$ | Undefined ca. | 2 primary lung ca. |
| 1 | Liver | D612$^-$ B72.3$^+$ | Undefined ca. | 1 met. colon ca. |
| Total 24 | | | | |

[a]Sampled by CT-FNA; [b]No. confirmed diagnoses/no. tested.

This clinical behaviour raises two main diagnostic problems in the management of colon-rectum carcinoma: (a) the identification of the colonic origin of metastases in patients bearing an occult primary tumour; (b) the correct diagnosis of recurrence in patients previously treated for a colon carcinoma.

Although serial determinations of serum CEA have been reported as an early sign of recurrence of colon carcinoma, this has been shown to be correct only in 58% of the patients (Eisman *et al.*, 1982). In addition, in patients with a cryptic primary tumour the measurement of CEA levels may be of little diagnostic value. This is due to the low tumour specificity of this marker.

Present radiological (Dixon *et al.*, 1981; Gianola *et al.*, 1984; Waneck *et al.*, 1984) and ultrastructural (Osamura *et al.*, 1985) methods to answer these questions still suffer from gross inaccuracy due to their lack of tumour specificity, or are often not performed on a routine basis.

Although the cytopathological analysis either of metastatic effusions or of FNA of non-palpable lesions detected on CT-scan represents a useful diagnostic tool for the staging and monitoring of patients bearing a colon-rectum cancer, it requires considerable experience and a correct morphological diagnosis of tumour origin, which can be made only in the clinical context of a known primary tumour (Ghosh *et al.*, 1983; Orell & Dowling 1983; Hilborne *et al.*, 1985).

The application of immunocytochemical methods on different cellular substrates (effusions, fine needle aspirates) could be expected to contribute to an increase in the diagnostic accuracy of the conventional cytopathology in these areas by introducing both the specificity of an antigen–antibody reaction and an objective interpretation of the results.

Attempts in this context have been only partially successful because of the lack of highly tumour specific antibodies (To *et al.*, 1982; Ramaekers *et al.*, 1984; Kyrkou *et al.*, 1985; Johnston *et al.*, 1986). Ideal reagents in this context should be capable of recognising a tumour associated antigen (TAA) endowed with: (a) high colon cancer specificity; (b) homogeneous expression unrelated to various degrees of tumour differentiation; (c) homogenous expression in metastatic foci independently from their anatomical distribution. Because the high molecular weight TAA recently identified by the murin MoAb D612 appears to fulfil most of these requirements we have employed this antibody in immunocytochemical assays aimed at evaluating whether this reagent is capable of increasing the accuracy of cytodiagnosis of colon-rectum cancer. Because this differentiation antigen is also expressed by about 20% of mucinous ovarian, lung, breast and gastric primary carcinomas, in order to increase the diagnostic accuracy of MoAb D612 this reagent has been employed together with other MoAb which have already been shown to possess a high diagnostic value (Mottolese *et al.*, 1988) when used in combination in detecting breast, lung and ovarian tumours. Otherwise MoAb D612, when tested on 60 effusions metastatic from breast, lung and serous ovarian carcinomas, was negative in 95% of the instances, thus demonstrating a restricted reactivity to primary and metastatic colon-rectum carcinomas. Two clinical features of colon-rectum cancer bearing patients should be recalled. First, as reported by different authors, more than 30% of all metastatic ovarian tumours are actually metastases from colon-rectum carcinomas (Harcourt & Dennis, 1968; Mazur *et al.*, 1984) and unsuspected intestinal tumours are often found in women presenting an ovarian mass initially diagnosed as a primary epithelial ovarian neoplasia (Morrow & Enker, 1984; Lash & Hart, 1987). In these clinical cases CT-guided FNA of ovarian tumours offers an accurate and relatively atraumatic alternative to surgical biopsy in many instances (Sevin & Nedji, 1983) avoiding time-delay and cost of surgery and allowing an adequate treatment of the patient immediately. However, FNA cytology of pelvic tumours is frequently difficult and it is often impossible to establish whether the specimens derive from a primary or metastatic tumour only on the basis of the morphological picture (Linsk & Franzen, 1983). Primary ovarian carcinomas, in some instances, can display the same cytological appearance as a metastatic adenocarcinoma. Furthermore, patients previously treated for a colon-rectum tumour are known to be at high risk to develop a second neoplasia of mammary, endometrial and pulmonary origin (Cahan *et al.*, 1974; Sugarbaker *et al.*, 1985). The results of the present study which employed MoAb D612 on different cytological specimens have shown that the addition of this reagent to a number of selected MoAb may help to increase the accuracy of this diagnostic panel of reagents by: (a) identifying the colonic origin of recurrences and of metastases in patients with an occult primary tumour; (b) detecting the presence of a second neoplasia in patients with a previous history other than colon cancer; (c) providing a differential diagnosis between primary ovarian tumour and ovarian metastases from a colon-rectum.

The addition to this diagnostic panel of MoAb recognising mucinous ovarian tumours such as those described recently by Sakakibara *et al.* (1988) may allow the differential diagnosis between metastatic mucinous colon carcinoma to the ovary from primary mucinous ovarian tumours. Furthermore, the lack of reactivity of MoAb D612 with prostatic cancer (Muraro *et al.*, 1989), although not evaluated immunocytochemically in the present study, is also likely to allow a differential diagnosis between colon and prostate tumours.

In conclusion, these findings clearly indicate that MoAb D612 may help to improve the diagnosis of colon carcinoma in different clinical situations as shown by the two clinical cases reported, thus allowing the choice of adequate therapeutic strategies of this neoplasia, which represents one of the most common internal malignancies in both sexes.

Supported by Progetto Finalizzato CNR, by AIRC, by the Italian Ministry of Public Health and by Tecnobiomedica. The authors wish to thank Dr J. Schlom of the NCI Bethesda for providing MoAb D612 and for his continuous support. Preliminary findings of this study were presented at the International Congress of Gastroenterology and Digestive Endoscopy Rome, 4–10 September 1988 and Asco Meeting, San Francisco, May 1989.

### References

AVRAMEAS, S. & TERNYNCK, T. (1969). The cross-linking of proteins with gluteraldehyde and its use for the preparation of immunoabsorbents. *Immunochemistry*, **6**, 53.

BAST, R.C., FEENEY, M., LAZARUS, H., NADLER, L.M., COLVIN, R.B. & KNAPP, R.C. (1981). Reactivity of a monoclonal antibody with human ovarian carcinoma. *J. Clin. Invest.*, **68**, 1331.

BERNARDINO, M.E. (1987). New diagnostic techniques in hepatic mass detection. In *Biology and Treatment of Colorectal Cancer Metastasis*, Mastromarino, A. (ed.) Martinus Nijhoff: Dordrecht.

CAHAN, W.G., CASTRO, E.B. & HAJDU, S.L. (1974). The significance of a solitary lung shadow in patients with colon carcinoma. *Cancer*, **33**, 414.

CANEVARI, S., FOSSATI, G., BALSARI, A., SONNINO, S. & COLNAGHI, M.I. (1983). Immunochemical analysis of the determinant recognized by a monoclonal antibody (MBrl) which specifically binds to human mammary epithelial cells. *Cancer Res.*, **43**, 1201.

COLCHER, D., HORAN-HAND, P., NUTI, M. & SCHLOM, J. (1981). A spectrum of monoclonal antibodies reactive with human mammary tumors. *Proc. Natl Acad. Sci. USA*, **78**, 3199.

DIXON, A.K., KELSEY FRY, I., MORSON, C.B. *et al.* (1981). Preoperative computed tomography of carcinoma of the rectum. *Br. J. Radiol.*, **54**, 655.

EISMAN, B., ROBINSON, W.A. & STEELE, G. (1982). *Follow-up of the Cancer Patient*. Thieme Verlag: New York.

GHOSH, A.K., SPRIGGS, A.I., PAPADIMITRIOU, T. & MASON, D.Y. (1983). Immunocytochemical staining of cells in pleural and peritoneal effusions with a panel of monoclonal antibodies. *J. Clin. Pathol.*, **36**, 1154.

GIANOLA, F.J., DWYER, A., JONES, A.E. & SUGARBAKER, P.H. (1984). Prospective studies of laboratory and radiologic tests in the management of colon and rectal cancer patients: I. Selection of useful preoperative tests through an analysis of surgically occult metastases. *Dis. Col. Rect.*, **27**, 811.

HARCOURT, K.F. & DENNIS, D.L. (1968). Laparotomy for 'ovarian tumors' in unsuspected carcinoma of the colon. *Cancer*, **21**, 9244.

HILBORNE, L.H., CHENG, L., NIEBERG, R.K. & LEWIN, K.S. (1985). Evaluation of an antibody to human milk fat globule antigen in the detection of metastatic carcinoma in pleural, pericardial and peritoneal fluids. *Acta Cytol.*, **30**, 245.

HSU, S.M., RAINE, L. & FANGER, H. (1981). Use of avidin-biotin-peroxidase complex (ABC) in immunoperoxidase techniques. A comparison between ABC and unlabeled antibody (PAP) procedures. *J. Histochem. Cytochem.*, **29**, 577.

JOHNSTON, W.W., SZPAK, C.A., LOTTICH, S.C., THOR, A. & SCHLOM, J. (1986). Use of a monoclonal antibody (B72.3) as a novel immunocytochemical adjunct for the diagnosis of carcinoma in fine needle aspiration biopsy specimens. *Hum. Pathol.*, **17**, 501.

JOHNSTON, W.W., SZPAK, C.A., LOTTICH, S.C., THOR, A. & SCHLOM, J. (1985). An adenocarcinoma associated determinant in human effusions: use of monoclonal antibody as an immunocytochemical adjuvant to diagnosis. *Cancer Res.*, **5**, 1894.

KYRKOU, K.A., IATRIDIS, S.G., ATHANISSIADOU, P.P., EMMANOULIDOU, A.G. & ATHANASSIADIS, P.P. (1985). Detection of benign and malignant origin of ascites with combined indirect immunoperoxidase assays of CEA and lysozyme. *Acta Cytol.*, **29**, 57.

LASH, R.H. & HART, W.R. (1987). Intestinal adenocarcinoma metastatic to the ovaries: a clinical pathologic evaluation of 22 cases. *Am. J. Surg. Pathol.*, **11**, 114.

LINSK, J.A. & FRANZEN, S. (1983). Abdominal aspiration. In *Clinical Aspiration Cytology*, Linsk, J.A. & Franzen, S. (eds) p. 169. J.B. Lippincott: Philadelphia.

MARTIN, S.E., MOSHIRI, S., THOR, A., VILASI, V., CHUE, E.W. & SCHLOM, J. (1986). Identification of adenocarcinoma in cytospin preparations of effusions using monoclonal antibody B72.3. *Am. J. Clin. Pathol.*, **86**, 10.

MAZUR, M.T., HSUEH, S. & GERSELLE, D.J. (1984). Metastases to the female genital tract. *Cancer*, **53**, 1978.

MENARD, S., RILKE, F., DELLA TORRE, G. & 5 others (1985). Sensitivity enhancement of the cytologic detection of cancer cells in effusions by monoclonal antibodies. *Am. J. Clin. Pathol.*, **83**, 571.

MIOTTI, A., CANEVARI, S., MENARD, S. & 6 others (1987). Characterization of human ovarian carcinoma associated antigen defined by novel monoclonal antibodies with tumor restricted specificity. *Int. J. Cancer*, **39**, 297.

MITCHELL, H.G. (1987). The evaluation of serial marker measurement for monitoring patients at risk of recurrent cancer: application to colorectal cancer. In *Biology and Treatment of Colorectal Cancer Metastasis*, Mastromarino, A. (ed.). Martinus Nijhoff: Dordrecht.

MORROW, M. & ENKER, W.E. (1984). Late ovarian metastases in carcinoma of the colon rectum. *Arch. Surg.*, **119**, 1385.

MOTTOLESE, M., VENTURO, I., PERRONE DONNORSO, R., GALLO CURCIO, C., RINALDI, M. & NATALI, P.G. (1988). Use of selected combinations of monoclonal antibodies to tumor associated antigens in the diagnosis of neoplastic effusions of unknown origin. *Eur. J. Cancer Clin. Oncol.*, **24**, 1277.

MURARO, R., NUTI, M., NATALI, P.G. & 6 others (1989). A monoclonal antibody (D612) with selective reactivity for malignant and normal gastrointestinal epithelium. *Int. J. Cancer*, **43**, 598.

NOUWEN, E.J., POLLET, D.E., EERDEKENS, M.W., HENDRIX, P.G., BRIER, T.W. & DE BROE, M.E. (1986). Immunohistochemical localization of placental alkaline phosphatase, carcinoembryonic antigen, and cancer antigen 125 in normal and neoplastic human lung. *Cancer Res.*, **46**, 866.

ORELL, S.R. & DOWLING, K.D. (1983). Oncofetal antigens as tumor markers in the cytologic diagnosis of effusions. *Acta Cytol.*, **30**, 625.

OSAMURA, R.Y., WATANABE, K. & AKATSUKA, Y. (1985). Peroxidase labeled antibody staining for carcinoembryonic antigen of cytologic specimens for light and electron microscopy. *Acta Cytol.*, **29**, 254.

RAMAEKERS, F., HAAG, D., JAP, P. & VOOIJS, P.G. (1984). Immunochemical demonstration of keratin and vimentin in cytologic aspirates. *Acta Cytol.*, **28**, 385.

RUSSO, C., CALLEGARO, L., LANZA, E. & FERRONE, S. (1983). Purification of IgG monoclonal antibody by caprylic acid precipitation (letter). *J. Immunol. Methods*, **65**, 269.

SAKAKIBARA, K., UEDA, R., OHTA, M., NAKASHIMA, N., TOMODA, Y. & TAKAHASHI, Y. (1988). Three novel mouse monoclonal antibodies, OM-A, OM-B, and OM-C, reactive with mucinous type ovarian tumors. *Cancer Res.*, **48**, 4639.

SEVIN, B.U. & NADJI, M. (1983). Pelvic fine needle aspiration cytology in gynecology. In *Clinical Aspiration Cytology*, Linsk, J.A. & Franzen, S. (eds) p. 21. J.B. Lippincott: Philadelphia.

SUGARBAKER, P.H., GUNDERSON, L.L. & WITTERS, R.E. (1985). Colorectal cancer. In *Cancer: Principles and Practice of Oncology*, 2nd edn, De Vita, V.T. Jr, Hellman, S. & Rosemberg, S.A. (eds) p. 866. J.B. Lippincott: Philadelphia.

SZPAK, C.A., JOHNSTON, W.W., LOTTICH, S.C., KUFE, D., THOR, A. & SCHLOM, J. (1985). Patterns of reactivity of four novel monoclonal antibodies (B72.3, DF3, B1.1 and B6.2) with cells in human malignant and benign effusions. *Acta Cytol.*, **28**, 356.

THOR, A., OHUCHI, N., SZPAK, C.A., JOHNSTON, W.W. & SCHLOM, J. (1986). Distribution of oncofetal antigen tumor-associated glycoprotein-72 defined by monoclonal antibody B72.3. *Cancer Res.*, **46**, 3118.

TO, A., DEARNALEY, D.P., ORMEROD, G., CANTI, G. & COLEMAN, D.V. (1982). Epithelial membrane antigen its use in the cytodiagnosis of malignancy in serous effusions. *Am. J. Clin. Pathol.*, **77**, 214.

VARKY, N.M., REISFELD, R.A. & WALKER, L.E. (1984). Antigens associated with a human lung adenocarcinoma defined by monoclonal antibodies. *Cancer Res.*, **44**, 681.

WANECK, R., LECHNER, G., JANTSCH, H., KOVATS, E. & SCHIESSEL, R. (1984). Lateral distant view for improved accuracy in locating rectal tumors. *Am. J. Radiat.*, **142**, 519.